# The Accuracy of Open-Tray vs. Snap on Impression Techniques in A 6-Implant Model: An In Vitro 3D Study

**DOI:** 10.3390/ma15062103

**Published:** 2022-03-12

**Authors:** Adi Arieli, Maram Adawi, Mahmoud Masri, Evgeny Weinberg, Ilan Beitlitum, Raphael Pilo, Shifra Levartovsky

**Affiliations:** 1 Department of Oral Rehabilitation, The Maurice and Gabriela Goldschleger School of Dental Medicine, Sackler Faculty of Medicine, Tel Aviv University, Tel Aviv 6997801, Israel; dr.arieli@gmail.com (A.A.); maram.adawi@gmail.com (M.A.); mahmoudmasri14@gmail.com (M.M.); 2 Department of Periodontology and Dental Implantology, Maurice and Gabriela Goldschleger School of Dental Medicine, Tel Aviv University, Tel Aviv 6997801, Israel; evgenywein@gmail.com (E.W.); beilan1612@gmail.com (I.B.); 3 Department of Oral Biology, The Maurice and Gabriela Goldschleger School of Dental Medicine, Sackler Faculty of Medicine, Tel Aviv University, Tel Aviv 6997801, Israel; rafipilo@gmail.com

**Keywords:** impression, 3D, closed-tray, open-tray, snap on

## Abstract

To compare the three-dimensional accuracy of an open-tray and two snap on impression techniques (with and without connecting the plastic caps of the snap on impression transfers) in a full arch 6-implant model, a reference acrylic resin model of the maxilla with six implants was fabricated. Prominent geometrical triangles, in the palate area, served as reference points for a digital overlap between scans. Three impression transfer techniques were evaluated and compared: open-tray direct impression (DI), snap on impression (SpO), and connected snap on impression (SpOC). Polyether impression material was used to make 30 impressions (n = 10), and the master model and all casts were digitally scanned with a laboratory optical scanner. The obtained 3D data were converted and recorded as STL files, which were imported to a 3D inspection software program. Angular deviations (buccal, occlusal and interproximal planes) between the study casts and the reference model were measured. Data were analyzed using one-way analysis of variance (ANOVA) and Tukey post hoc test, with 0.05 used as the level of significance. The 3D angular deviations from the master model revealed no significant differences between the DI and SpO impression groups, but there were significant differences in the SpOC impression group, particularly in the buccal and occlusal planes. In all groups, the 3D angular deviation between the most distal scan abutments on each side of the model was significantly different from all other areas when compared to the master model. Within the limits of this study, it is possible to conclude that the indirect closed tray snap on impression technique with unconnected plastic caps exhibited the same three-dimensional accuracies as the direct open tray technique. The indirect closed tray snap on impression technique with connected plastic caps was less accurate than either the indirect closed tray snap on impression technique with unconnected plastic caps or the direct open tray technique. In the case of full arch implant supported prostheses, inaccuracies may be expected in the most distal implants for all the three impression techniques evaluated in this study. Further in vitro and in vivo research is required.

## 1. Introduction

The procedure of making an impression is a crucial stage in the implant prosthodontic treatment process, as it directly affects the accuracy of the definitive cast and the passive fit of the implant supported superstructure [1]. A misfit of the implant supported prosthesis leads to mechanical and biological complications [2,3] since, unlike natural teeth, dental implants lack the cushioning effect of periodontal fibers and cannot completely compensate for any imprecision in the superstructure [4]. Although, practically speaking, the achievement of a perfect passive fit is almost impossible, aspiring to diminish impression errors and improve the outcome of the impression procedure is an important part of implant prosthodontic treatment. A number of studies have investigated the clinical factors that affect the precision of implant supported impressions. These include studies comparing open and closed-tray impression techniques [5], or the use of different impression materials [6]. Still, other studies have examined the effect of connecting the implant transfers on the accuracy of the impressions [7,8], as well as the effect of implant angulation or the design of different prosthetic parts [9].

There are currently two main conventional implant supported impression techniques: the direct technique that uses an open-tray; and the indirect technique that uses a closed-tray [10]. While the direct impression technique is considered to be more accurate [6,11,12], the indirect technique is easier to use and more comfortable for the patient, especially for those with a strong gag reflex or limited mouth opening [9,13,14,15]. Unfortunately, the need to reposition the transfer in the indirect closed-tray technique can produce less predictable results than the pickup of the transfers that are used in the direct open-tray technique [16]. In order to resolve this issue, the implant manufacturers have introduced snap on plastic impression caps, where the plastic cap is picked up with the closed-tray impression, thereby, simplifying the procedure and improving the accuracy [5,13,17]. The indirect snap on technique is easy to use, time saving, and more comfortable for both patient and clinician, especially for full arch cases.

Previous studies have reported that the level of accuracy achieved by the snap on impression method is similar or even higher than achieved by the direct impression method [4,17,18]. Conversely, the results of other studies indicated that the direct impression technique provides better accuracy than the indirect snap on technique [19,20]. One study that used four unilateral implant analogs for each cast to compare the accuracy of casts produced by snap on versus direct impression, reported a similar dimensional accuracy for the two techniques [17]. Another study made a similar comparison between the techniques by using two implant analogs on each side of casts that were placed symmetrically to the midline, and also concluded that there was no difference in accuracy between the snap on and open-tray techniques [4]. 

Most clinicians now agree that the direct technique with splinting of impression copings provides greater accuracy than the non-splinted option, although, other studies have reported no significant differences in splinted versus non-splinted direct methods [1,5,21]. Papaspyridakos et al. reported in a systematic review that for completely edentulous patients, 22 in-vitro studies and three clinical studies support the splinting technique [21]. 

With the increasing popularity of digital dental technology, in-vitro studies have compared traditional impression procedures with digital impression approaches [22]. While digital impression accuracy was shown to be clinically acceptable for the fabrication of single implant-supported crowns and short FDPs, there is still a lack of consensus regarding the accuracy and clinical acceptability of digital versus conventional methods for full-arch impressions [23,24]. 

To date, very few studies have examined the 3D accuracy of the closed-tray snap on versus the open-tray impression techniques in a full arch model. Moreover, no study has evaluated the impact of connecting the plastic caps on the accuracy of the closed-tray snap on impression technique. 

The aim of the current study was to compare the three-dimensional accuracy of open-tray and two closed-tray snap on impression techniques (with and without connecting the plastic caps of the snap on impression transfers) in a full arch 6-implant model. The null hypothesis was that there would be no significant differences between the accuracy of these three impression techniques.

## 2. Experimental Section

**Figure 1:**
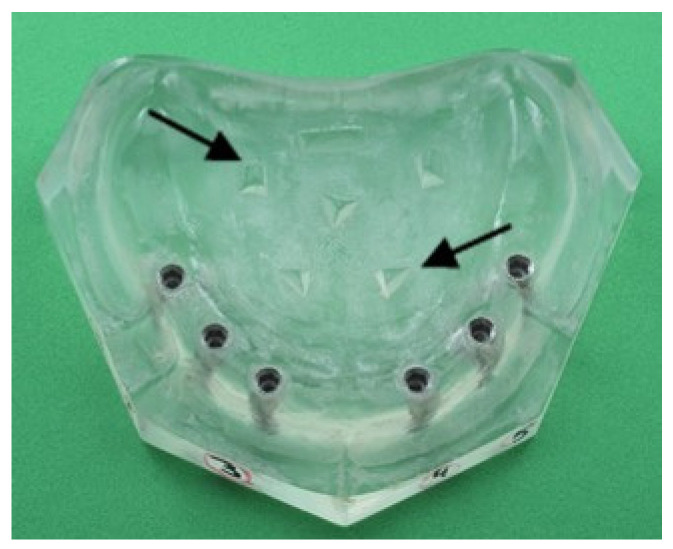
Master model with six analogs located in bilateral symmetry to the midline.

A custom master model representing a clinical situation of an edentulous maxilla was fabricated from chemically cured methyl-methacrylate (Palpress, Heraus Kulzer GmbH, Wehrheim, Germany). Prominent geometrical triangles were marked in the palate area as reference points for the digital overlap between scans. A milling machine (Alliant Vertical Milling Machine, Alliant, Cincinnati, OH, USA) was used to prepare six parallel, 12 mm long and 4.5 mm in diameter, implant sockets. The holes were located in bilateral symmetry to the midline, with a distance of 10 mm between them. Six implant analogs (MD-RSM10 3.75Ø, MIS/Divident, Or-Yehuda, Israel) were attached to the holes using the same chemically cured methyl-methacrylate (Figure 1). 

**Figure 2:**
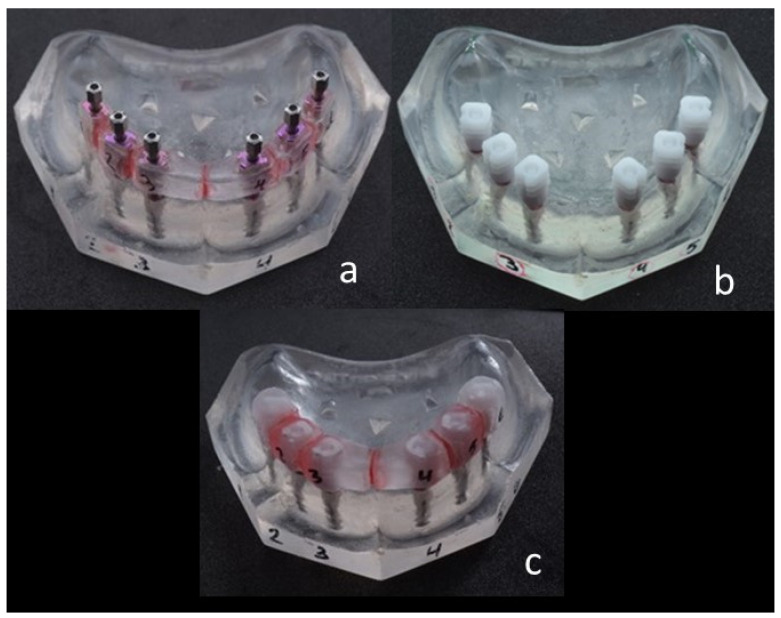
Study groups: (**a**)—direct impression group (DI); (**b**)—indirect snap on impression group (SpO); (**c**)—indirect connected snap on impression group (SpOC).

The parallelism of the six analogs was ensured by a surveyor (Surveyor B2 Bio-art). Three study groups were included in this study: the direct impression group (DI-direct impression, Figure 2a), the indirect snap on impression group (SpO, Figure 2b), and the indirect snap on impression group in which the plastic caps were connected to each other (SpOC, Figure 2c). 

Open-tray impressions of the master model were made for the DI group using custom chemically cured acrylic trays (COE TRAY PLASTIC™, GC America, Alsip, IL, USA) and suitable impression transfers (MIS MD-I0375, MIS/Divident, Or-Yehuda, Israel). The impression transfers were screwed to the analogs with 10 Ncm torque, as recommended by the manufacturer, and were connected to each other with transparent self-curing acrylic resin (Palapresss, Heraus Kulzer GmbH, Germany) 1 day prior to impression-making. In order to avoid polymerization shrinkage, the connections were thinly sectioned and the transfers were reattached with a thin layer of self-curing acrylic material (Pattern resin LS, GC America, Alsip, IL, USA). One can notice the thin layer of the Pattern resin due to its different color from the transparent acrylic resin. 

Closed-tray impressions of the master model were made for the SpO and SpOC groups, using stock metal trays and snap on impression transfers (MIS CS-IT300 MIS/Divident, Or-Yehuda, Israel). The metal parts of the transfers were screwed to the analogs with 10 Ncm torque, according to the manufacturer’s recommendations. The plastic impression caps were pushed on top of the metal parts until they clicked to ensure a secure fit. The plastic impression caps in the SpOC group samples were connected to each other in the same way as for the DI group. 

Ten impressions of the master model were taken for each study group, using a one-step monophase technique, with polyether as the impression material (Impregum, Penta soft, 3M ESPE Dental, Medizin, Germany). This gave a total of 30 impressions. Following the manufacturer’s instructions, the impression material was allowed to polymerize for 6 min and was then removed from the master model. For the samples in the SpO and SpOC groups, the plastic caps were then picked up and embedded in the impression, while for the DI group, it was necessary to unscrew the transfers in order to loosen them before the impression was removed. 

Implant analogs (MIS MD-RSM10 3.75Ø, MIS/Divident, Or-Yehuda, Israel) were then snapped into all the transfers and the impressions were poured. The material used was Type IV dental stone (Silky Rock, Whipmix, Louisville, KY, USA) that was mixed in a vacuum mixer (Multiwac, Degusa, Germany), according to the manufacturer’s instructions (100 mg powder/30 mL water). The master model and all study casts were digitally scanned with a laboratory optical scanner (Zirkon Zahn ARTI Scanner S600, Woodstock, GA, USA) using scan bodies (Scanning Abutment MIS, MIS/Divident, Or-Yehuda, Israel) that were attached to the analogs with 10 Ncm torque, as recommended by the manufacturer. The scan bodies were attached in the same position on the master model as well as on the study models in order to avoid deviations. 

**Figure 3:**
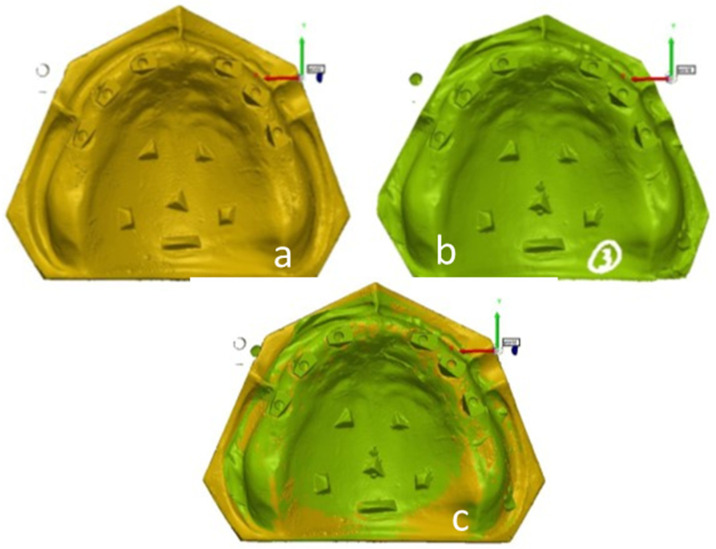
(**a**)—master model scan; (**b**)—study group scan; (**c**)—master model scan digitally overlapped with study group scan.

All obtained 3D data were converted and recorded as standard tessellation language (STL) files, which were imported into a reverse engineering software (PolyWorks InspectorTM, InnovMetric Software, Vétroz, VALAIS, Canada) for superimposition and 3D deviation assessment. The STL files from the study models were digitally overlapped with the STL file from the master model, which served as the reference data. The superimposition was performed by using the predetermined reference points that were marked on the master model (Figure 3).

**Figure 4:**
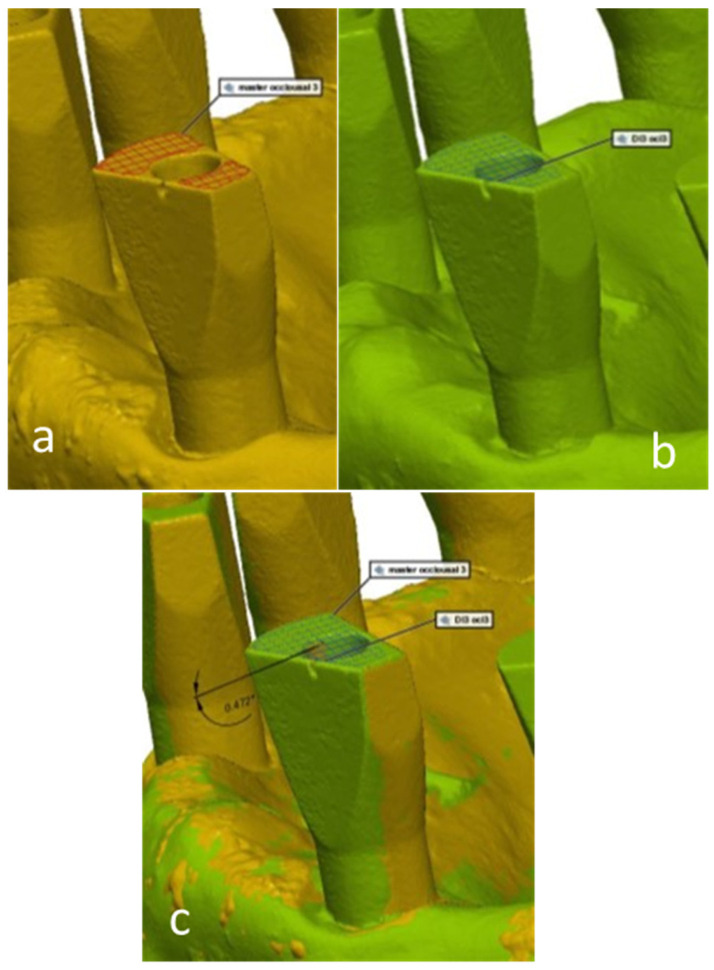
A digital 3D measurement of the angle between each matching scan abutment occlusal surface in the study model compared to the master model. (**a**)—master model scan; (**b**)—study group scan; (**c**)—master model scan digitally overlapped with study group scan.

**Figure 5:**
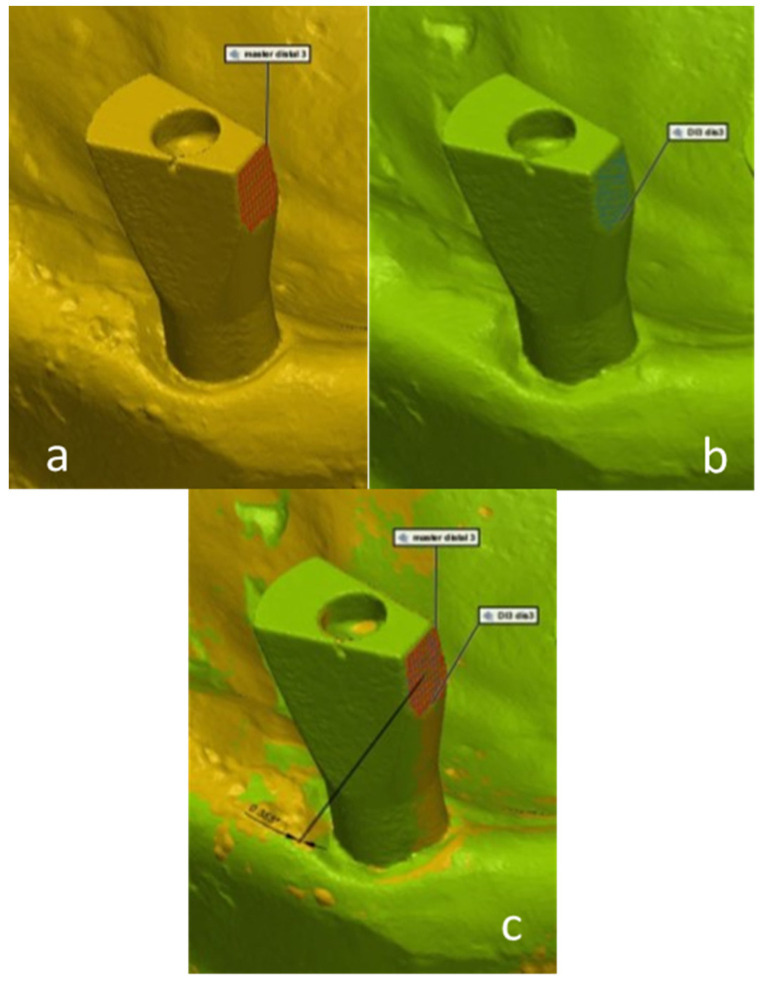
A digital 3D measurement of the angle between each matching scan abutment interproximal surface in the study model compared to the master model. (**a**)—master model scan; (**b**)—study group scan; (**c**)—master model scan digitally overlapped with study group scan.

**Figure 6:**
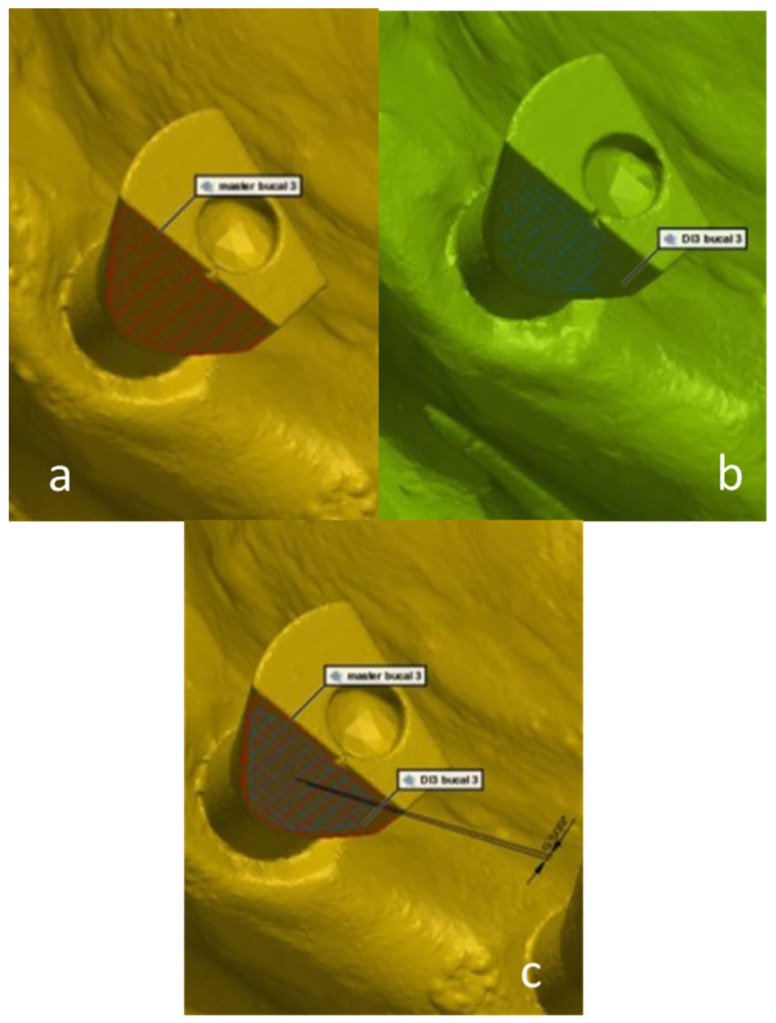
A digital 3D measurement of the angle between each matching scan abutment buccal surface in the study model compared to the master model. (**a**)—master model scan; (**b**)—study group scan; (**c**)—master model scan digitally overlapped with study group scan.

Three surfaces were defined on each scan body: buccal surface, occlusal surface, and interproximal surface. The 3D deviation measurements were made with the assistance of the computer software, by calculating the angle between each matching scan body surface in the study model compared to the master model. The values of the obtained angles reflect the inaccuracy between the master model and the study models in the three surfaces (Figure 4—occlusal; Figure 5—interproximal; Figure 6—buccal). 

**Figure 7:**
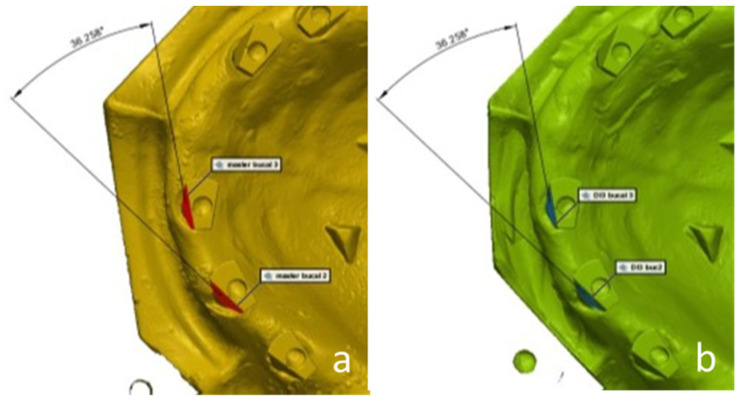
A digital measurement of the angle between the buccal surface of each scan abutment to the same surface on the adjacent scan abutment. (**a**)—master model scan; (**b**)—study group scan.

In addition, the accuracy of the position of each scan abutment to its neighbor was also examined on each study model and compared to the master model. For this purpose, each study model was divided into six areas, where area 1 addresses the accuracy between scan abutments 1 and 2 and proceeding to area 6, which considers the accuracy between scan abutments 6 and 1 (the most distally placed abutments). The angle between the buccal surface of each scan abutment to the same surface on the adjacent scan abutment was calculated digitally for all study models and then compared to the master model (Figure 7). 

The buccal surface was chosen as the reference surface for convenience because it has the largest surface on the scan abutment. 

### Statistical Methods and Synthesis of Results

Statistical analysis was conducted using a statistical software program (IBM SPSS Statistics, v22.0, IBM Corp, NY, USA). The values of the angles measured on each matching scan abutment surface on all three surfaces in the study model were compared to the master model by using one-way ANOVA. In order to determine which study group was different from the master model, Post hoc Tukey’s tests were used to examine differences between the study groups and the master model. ANOVA with repeated measures; within and between subject factor group and pairwise comparison Bonferroni’s method, were used to examine the accuracy of the position of each scan abutment relative to its neighbor. A significance level of *p* = 0.05 was used.

## 3. Results

The results of the one-way ANOVA analysis for all three surfaces of the study models and the master model are presented in Table 1. 

There were significant differences in the angle values in the occlusal (*p* = 0.000) and the buccal (*p* = 0.004) surfaces between all the study models, as compared to the master model. For the interproximal surface, the difference in the angle values showed a weak significance (*p* = 0.049). Post hoc Tukey’s tests revealed no significant differences between the angle values in the SpO group and the DI groups, for any of the three surfaces (Table 2).

Means for groups in homogeneous subsets are displayed.

**Figure 8:**
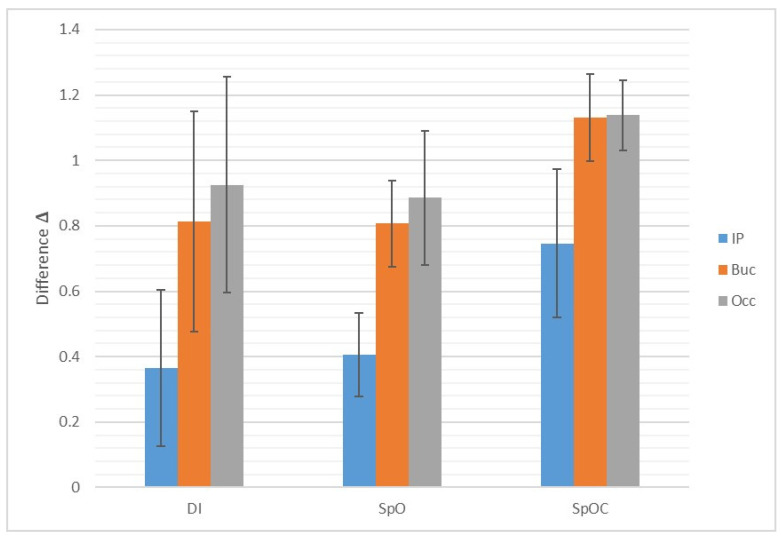
The difference between the measured angles in each study model compared to the master model for all surfaces. Occ-occlusal; Buc-buccal; IP-interproximal.

In contrast, there were strong significant differences between the angles of the occlusal and buccal surfaces of the SpOC group compared to the SpO group and the DI group, and a weak but acceptably significant difference between the angles of the interproximal surfaces (Figure 8).

**Figure 9:**
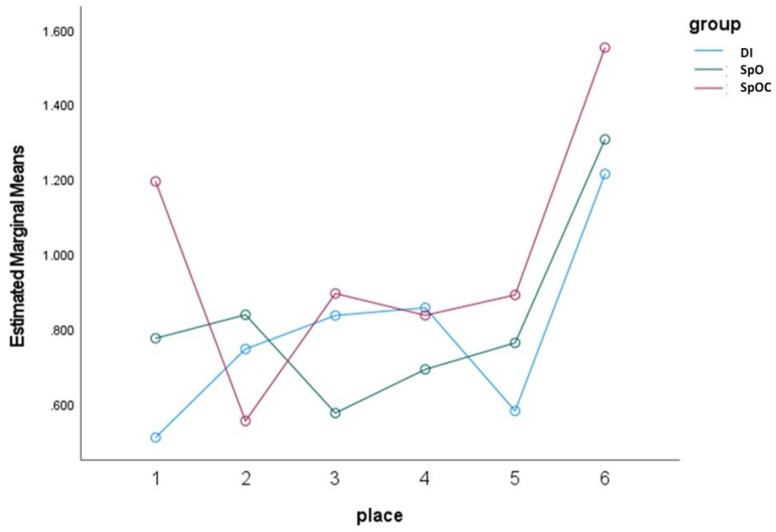
The accuracy of the position of each scan abutment to the adjacent one for the three study groups. DI—direct impression group; SpO—indirect snap on impression group; SpOC— indirect connected snap on impression group.

The results of ANOVA with repeated measures revealed that only area 6, which measures the accuracy in position between the most distally placed abutments, displayed significant differences in the angle values between the study model and the master model. This was true for all the study groups (Figure 9).

## 4. Discussion

Many attempts have been made to introduce novel impression techniques. Modified impression techniques using plastic snap on impression copings are very common nowadays. The objective of this study was to evaluate the three-dimensional accuracy of open-tray (DI) and two closed-tray snap on impression techniques, namely with (SpOC) and without connecting (SpO) the snap on, in a full arch 6-implant model. Computer software was used to calculate the angle between each matching scan body surface in the study model. A comparison between the values obtained and the corresponding angles in the master model provides a measure of the inaccuracies between the master and the study models. The null hypothesis was partially rejected. We found no significant differences between the SpO group and the DI groups in the angles of any of the three surfaces, but the results in the SpOC group were significantly different from the other two groups. This result suggests that the indirect closed-tray snap on impression technique with unconnected plastic caps (SpO) exhibits comparable three-dimensional accuracy to that of the direct open-tray (DI) technique. This is an important point since the DI technique has been extensively studied and found to be the most accurate method for implant impression technique, especially when the transfers were connected by acrylic material [1,2,16]. Our results, indicating that the indirect closed-tray snap on impression technique (SpO) is as accurate as the direct open-tray technique (DI), are also in accordance with the studies of Akca et al., Nakhaei et al. and Ozcelik et al. [4,17,25]. 

While our results revealed no significant difference in the angle values in the SpO group and the DI group for any of the three surfaces (Table 2), the angles in the SpOC group were significantly different. This finding suggests that the SpO technique is as accurate as the DI technique, as long as the plastic caps are not connected to each other. This is an important contribution, since to our knowledge, this study is the first to evaluate the influence of connecting the plastic caps on the accuracy of the closed-tray snap on impression technique. 

The significant difference between the SpO and SpOC study groups might be explained by the dimensional inaccuracy of the splinted plastic caps caused by acrylic resin shrinkage. However, the inaccuracy remained even when the connections were severed and the caps were reattached with the same acrylic material recommended for the DI technique [26]. Another option for the inaccuracies may be related to the flexibility of the plastic compartments used in the indirect closed-tray snap on impression technique, which may result in distortion if there is shrinkage of the attached acrylic resin. 

All the results shown in our study were statistically significant on all three tested surfaces, but on the interproximal surface, the significance was weaker albeit acceptable (Figure 8). This can be explained by the fact that the interproximal surfaces of the scan body possess the smallest surface area. The 3D inspection software program presents each surface as a collection of pixels derived from the digital scan in the STL file. Because of its narrow area, the number of pixels collected in each scan of the interproximal surface is rather low, which may affect the accuracy and thus weaken the statistical significance. 

Previous studies that compared the accuracy of different impression techniques used either digital scanning methods [25,27] or analog methods, employing a coordinate measuring machine (CMM), microscope, or strain gauge [9,12,28]. The most accurate results were obtained by digital methods as described by Ozcelik et al. [25] and Kurtulmus-Yılmaz et al. [27] who used an optical scanner to compare the long axes of the impression copings between the study groups and the master model. Our study is able to provide additional data because we used a laboratory optical scanner (Zirkon Zahn ARTI Scanner S600) and computer software (PolyWorks InspectorTM, Canada) to make a digital comparison for superimposition and 3D deviation assessment of each individual surface (buccal, occlusal, interproximal) of the scan body. These data enable us to obtain a more accurate and detailed three-dimensional perspective. 

While promising, there remains the question that the digital impression technique may still not be accurate enough for full arch implant cases, such as the 6-implant full arch master model examined here [23,29]. Other possible complications of the digital method include the observation that the accuracy of the digital impression depends on the implant scan body (ISB) material and position [30]. Moreover, digital workflow is still not a common practice in all dental practices worldwide. Thus, conventional impression methods, such as the snap on technique, are currently still required and widely practiced, especially in full arch implant supported cases.

Our examination of the accuracy of the position of adjacent scan abutments revealed that the angles in area 6, which measures the two most distal abutments, were significantly different from those in the master model, for all study groups (Figure 9). The measurements were made on the buccal surface of each scan body assuming that since it has the largest surface area it would be able to provide more pixels for a more accurate digital analysis. Our findings are in accordance with the results of Rech-Ortega et al. [31], who used a CMM analog device, and demonstrated that the distance between the distal analogs was significantly different for both conventional and digital impression techniques. Our support of their results with the use of a high precision digital scanner and 3D inspection software program, suggests that the highest inaccuracies in cases of full arch implant supported restoration are most likely to be found between the most distant analogs, regardless of the impression technique employed. 

Framework design and fabrication as well as impression accuracy are important for framework fit, but standardized values for acceptance of passive fit are lacking. Marginal discrepancies of 30 up to 150 μm between frameworks and abutments have been suggested as acceptable to prevent biological and technical complications [29]. However, there remains insufficient literature data on the discrepancies between the angles in the framework and the implant analogs that will provide an acceptable clinical fit, and further studies will be required.

As mesenchymal stem cells were already being shown to accelerate osseointegration with dental implants, other novel therapeutic modalities could be beneficial for future researchers in relation to different impression techniques [32].

Our results represent the first digital comparison of the three separate surfaces of a scan body in a master model of six vertically aligned analogs. The limitation of the current study was that it is an in vitro study, which cannot fully reproduce the clinical conditions. Other limitations are the small sample size and the use of only one implant company with parallel analogs. Different results of the impression accuracy may be obtained with the angulated placement of implants. More research is needed in order to verify our results on a model with tilted analogs, which more truthfully replicates the clinical situation. In addition, further in vivo research is required.

## 5. Conclusions

Within the limits of this in vitro study, it is possible to conclude that the indirect closed tray snap on impression technique with unconnected plastic caps exhibited the same three-dimensional accuracies as the direct open tray technique. The indirect closed tray snap on impression technique with connected plastic caps was less accurate than either the indirect closed tray snap on impression technique with unconnected plastic caps or the direct open tray technique. In the case of full arch implant supported prostheses, inaccuracies may be expected in the most distal implants for all the three impression techniques evaluated in this study. More research needs to be performed in the future in order to verify our results on a model with a variety of implant positions: tilted analogs, different heights and different platforms which are more truthfully replicate the clinical situation.

## Figures and Tables

**Table 1:** One-way ANOVA analysis between the three surfaces of the study and master models.

		Sum of Squares	df	Mean Square	F	Sig.
Occlusal	Between Groups	0.875	2	0.438	10.510	0.000
	Within Groups	1.124	27	0.042		
	Total	2.000	29			
Buccal	Between Groups	0.687	2	0.343	6.879	0.004
	Within Groups	1.348	27	0.050		
	Total	2.035	29			
Interprox	Between Groups	0.368	2	0.184	3.375	0.049
	Within Groups	1.471	27	0.054		
	Total	1.839	29			

**Table 2:** Post-hoc Tukey’s test comparing the three surfaces of each study group and the master model.

Group	N	Buccal Subset for alpha = 0.05	N	Occlusal Subset for alpha = 0.05	N	Interprox Subset for alpha = 0.05
	1	2	1	2	1	
Direct Impression (DI)	10	0.3663	10	0.8068	10	0.8855
Snap On Impression (SpO)	10	0.4050	10	0.8132	10	0.9255
Snap On Impression Connect (SpOC)	10	0.7464	10	1.1309	10	1.1378

## Data Availability

Data available on request due to restrictions of privacy. The data presented in this study are available on request from the corresponding author. The data are not publicly available due to privacy.
